# The Incidence and Prevalence of Diabetes Mellitus and Related Atherosclerotic Complications in Korea: A National Health Insurance Database Study

**DOI:** 10.1371/journal.pone.0110650

**Published:** 2014-10-16

**Authors:** Bo Kyung Koo, Chang-Hoon Lee, Bo Ram Yang, Seung-sik Hwang, Nam-Kyong Choi

**Affiliations:** 1 Department of Internal Medicine, Seoul National University College of Medicine, Seoul, Republic of Korea; 2 Department of Internal Medicine, Boramae Medical Center, Seoul, Republic of Korea; 3 Division of Clinical Epidemiology, Medical Research Collaborating Center, Seoul National University College of Medicine, Seoul, Republic of Korea; 4 Department of Preventive Medicine, Seoul National University College of Medicine, Seoul, Republic of Korea; 5 Department of Social and Preventive Medicine, Inha University School of Medicine, Incheon, Republic of Korea; 6 Institute of Environmental Medicine, Seoul National University Medical Research Center, Seoul, Republic of Korea; Institute of Endocrinology and Metabolism, Islamic Republic Of Iran

## Abstract

**Aims/Introduction:**

The incidence and prevalence of type 2 diabetes mellitus (T2DM) and related macrovascular complications in Korea were estimated using the Health Insurance Review and Assessment (HIRA) database from 2007–2011, which covers the claim data of 97.0% of the Korean population.

**Materials and Methods:**

T2DM, coronary artery disease (CAD), cerebrovascular disease (CVD), and peripheral artery disease (PAD) were defined according to ICD-10 codes. We used the Healthcare Common Procedure Coding System codes provided by HIRA to identify associated procedures or surgeries. When calculating incidence, we excluded cases with preexisting T2DM within two years before the index year. A Poisson distribution was assumed when calculating 95% confidence intervals for prevalence and incidence rates.

**Results:**

The prevalence of T2DM in Korean adults aged 20–89 years was 6.1–6.9% and the annual incidence rates of T2DM ranged from 9.5–9.8/1,000 person-year (PY) during the study period. The incidence rates of T2DM in men and women aged 20–49 years showed decreasing patterns from 2009 to 2011 (*P*<0.001); by contrast, the incidence in subjects aged 70–79 years showed increased patterns from 2009 to 2011 (*P*<0.001). The incidence rates of CAD and CVD in patients newly diagnosed with T2DM were 18.84/1,000 PY and 11.32/1,000 PY, respectively, in the year of diagnosis. Among newly diagnosed individuals with T2DM who were undergoing treatment for PAD, 14.6% underwent angioplasty for CAD during the same period.

**Conclusions:**

Our study measured the national incidences of T2DM, CAD, CVD, and PAD, which are of great concern for public health. We also confirmed the relatively higher risk of CAD and CVD newly detected T2DM patients compared to the general population in Korea.

## Introduction

The International Diabetes Federation (IDF) estimated the global prevalence of diabetes to be 151 million in 2000 [1] and 285 million in 2010 [2]. The IDF reported that 366 million people had diabetes in 2011, and this prevalence is expected to rise to 552 million by 2030 [3].

From 1970 to 2000, the prevalence of diabetes in South Korea increased about threefold [4], and 9–10% of Korean adults aged ≥30 years were affected by the disease in the early 2000s according to the Korea National Health and Nutritional Examination Survey (KNHANES) [4], [5]. The prevalence of diabetes is also increasing in other developing countries in Asia such as China [6]–[8] and India [9]. A recent meta-analysis reported that the prevalence of diabetes mellitus has significantly increased in China from 2.6% to 9.7% from 2000–2010 [10]. In developed countries such as Canada [11] and the US [12], the prevalence of diabetes has also continued to increase throughout the past decade. However, the prevalence in the Korean population remained stable over the same time period [13]. Furthermore, we recently reported that the prevalence of diabetes among women aged 30–59 years decreased trend from 2001 to 2010 [14]. It is important to investigate the incidence and prevalence of diabetes mellitus in the Korean population considering these trends in the occurrence of diabetes mellitus; however, current data are limited. In one community-based cohort study that included individuals in Korea between the ages of 40 and 79, the annual incidence of type 2 diabetes (T2DM) ranged from 1.33% to 5% [15].

The National Health Insurance (NHI) program in Korea was initiated in 1977 and achieved universal coverage of the population by 1989. The Health Insurance Review and Assessment (HIRA) service covers the claims of 97.0% of the population in Korea; those of the remaining 3% of the population are covered by the Medical Aid Program. Accordingly, the HIRA database contains information on almost the entirety of the insurance claims, including prescribed medications and procedures, for the Korean population of approximately 50 million [16]. The present study was performed to estimate the incidence and prevalence of T2DM and related macrovascular complications in the entire population of South Korea using the NHI claims database from 2007–2011.

## Methods

### Data collection

We used HIRA data recorded between 1 January 2007 and 31 December 2011. The HIRA database contains information on all insurance claims for about 97.0% of the population in Korea [16]. The HIRA service provided the data after de-identification, and the data included age, gender, diagnosis, date of hospital visits, drug prescriptions received during inpatient and outpatient visits, hospital admissions, medical procedures, and emergency department visits. Drug information included the brand name, generic name, prescription date, and duration and route of administration. Diagnoses were coded according to the International Classification of Disease (10th revision; ICD-10). The study was approved by the Boramae Medical Center Institutional Review Board.

### Study population

**Figure 1:**
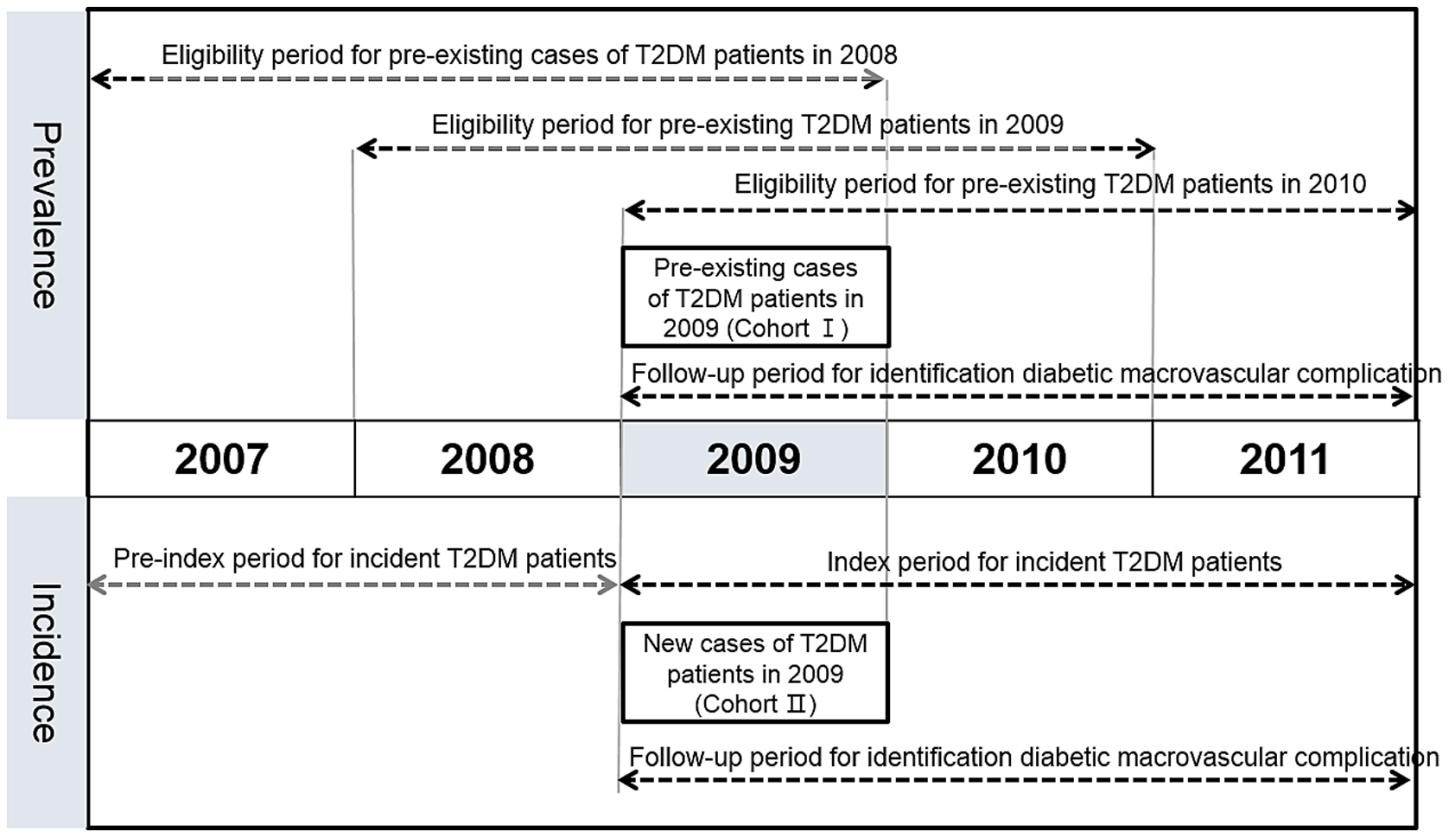
Schematic description of the study period.

The prevalence of T2DM patients aged 20–89 years from 2008 to 2010 was defined according to the following eligibility criteria: the presence of (1) at least two claims per year under ICD-10 codes E11–14 or (2) at least one claim per year for the prescription of anti-diabetic medication (under ICD-10 codes E11–14) [17]. Anti-diabetic medications included insulin, sulfonylureas, metformin, thiazolidinediones, α-glucosidase inhibitors, and meglitinides. We excluded patients with type 1 diabetes mellitus, defined as those who (i) were prescribed insulin without oral anti-diabetic agents and (ii) had at least one claim under the ICD-10 code E10, without the code E11, from 2007 to 2011 (**Figure 1**).

The incidence of T2DM from 2009 to 2011 was defined in individuals who met either of the selection criteria, (1) or (2), for estimating the prevalence of T2DM. We subsequently excluded those with preexisting diabetes two or more years before the study period, which included patients who were diagnosed with any type of diabetes mellitus (E10–14) or prescribed any anti-diabetic medications from 1 January 2007 to 31 December 2008 (**Figure 1**). Accordingly, the remaining cases had at least two years and up to five years of disease-free status before the index date and were regarded as the new cases of T2DM. The date of the earliest claim regarding T2DM on or after 1 January 2009 was defined as the index date. The index date was regarded as the incident time, and the patient was counted as a new case in that year.

### Case definition of cardiovascular and cerebrovascular disease

To investigate the incidence and prevalence of macrovascular complications in subjects with T2DM, we constructed sub-cohorts of new cases of T2DM and pre-existing cases of T2DM in 2009 (**Figure 1**). The incidence and prevalence, from 2009 to 2011, of macrovascular diabetic complications such as coronary artery disease (CAD), cerebrovascular disease (CVD), and peripheral artery disease (PAD), and amputation were analyzed within the sub-cohorts. CAD and CVD were identified by the following criteria, respectively: (1) medical claim(s) (including all inpatient hospital, outpatient hospital, medical visit, and emergency room claims) with the corresponding disease as the principal diagnosis, or additional diagnosis with an ICD-10 diagnosis code, or procedure code (**Table S1** in File S1) and (2) medical claim(s) regarding any percutaneous intervention or surgery associated with the corresponding disease. We used the Healthcare Common Procedure Coding System codes provided by the HIRA service to identify the associated procedure or surgery (**Table S1** in File S1). Since previous studies showed that the current diagnostic coding system is less sensitive to and less specific for PAD-related illness and treatment than for CAD or CVD [18], [19], we did not use ICD-10 diagnosis codes for defining PAD; instead, we defined PAD using claims regarding any percutaneous intervention or surgery associated with PAD (**Table S1** in File S1). Amputation of lower extremities was identified by the medical claim(s) that included an associated procedure or surgery code (**Table S1** in File S1). To detect new cases of CAD, CVD, and PAD in each cohort year, we eliminated all cases with a claim for each corresponding disease that occurred before the cohort year. Hospital admissions for CAD, CVD, and PAD were measured using inpatient claims with the principal diagnosis of the corresponding disease.

### Statistical analysis

All data were analyzed using SAS software version 9.3 (SAS Institute, Inc., Cary, NC, USA). Age- and gender-specific annual prevalence rates of T2DM from 2008 to 2010 were calculated by dividing the number of T2DM patients by the Korean population from the 2010 Population and Housing Census (**Table S2** in File S1) [20]. The age- and gender-specific annual incidence rate (number of new cases in each year/number of individuals at risk), and the person-time incidence rate, for 2009–2011, were calculated. The number of individuals at risk in each year was calculated using the following equation [the total population from the 2010 Census – (number of pre-existing cases in the previous year + half of the number of new cases in the year)]; this equation which assumes that new cases developed in the middle of a year. A Poisson distribution was assumed for calculating 95% confidence intervals (CIs) for rates of prevalence and incidence. The annual trends in prevalence and incidence were tested using Poisson regression within age–gender strata. Additionally, Poisson regression was used to analyze the annual trends in prevalence and incidence, with adjustment for age and gender.

## Results

### Prevalence and incidence of type 2 diabetes mellitus

There were 2,224,876 cases in 2008, 2,368,587 cases in 2009, and 2,516,350 cases in 2010 that fulfilled the study criteria for T2DM and were included in the final analysis. The prevalence of T2DM was 6.1% in 2008, 6.5% in 2009 and 6.9% in 2010 (6.4–7.3% in men and 5.7–6.4% in women from 2008–2010) (**Table 1**). With adjustment for age and gender, annual prevalence increased from 2008 to 2010 (*P*<0.0001). In men aged 60–79, the prevalence of T2DM increased each year and reached 19.2–20.0% in 2010 (*P*<0.0001). In women, the prevalence of T2DM was substantially lower than in men of same age in subjects aged <60 years; notably, women aged 30–49 years were shown to have about half the prevalence of T2DM than men of the same age (**Table 1**).

**Table 1:** The prevalence of type 2 diabetes mellitus in the Korean population aged 20–89 years.

		Year								*P*-value*
		2008		2009		2010		2008–2010		
		N	Prevalence % (95% CI)	N	Prevalence % (95% CI)	N	Prevalence % (95% CI)	N	Prevalence % (95% CI)	
Total		2,224,876	6.1 (6.1–6.1)	2,368,587	6.5 (6.4–6.5)	2,516,350	6.9 (6.9–6.9)	3,079,126	8.4 (8.4–8.4)	<0.0001
Men	Total	1,152,366	6.4 (6.4–6.4)	1,233,009	6.9 (6.9–6.9)	1,315,234	7.3 (7.3–7.3)	1,605,387	8.9 (8.9–9.0)	<0.0001
	20–29	8,379	0.2 (0.2–0.2)	8,592	0.3 (0.2–0.3)	8,382	0.2 (0.2–0.2)	14,936	0.4 (0.4–0.4)	0.982
	30–39	57,920	1.5 (1.5–1.5)	58,464	1.5 (1.5–1.5)	57,000	1.5 (1.4–1.5)	87,496	2.2 (2.2–2.2)	0.007
	40–49	220,675	5.4 (5.3–5.4)	229,411	5.6 (5.6–5.6)	228,034	5.5 (5.5–5.6)	308,762	7.5 (7.5–7.5)	<0.0001
	50–59	341,748	10.5 (10.5–10.6)	369,824	11.4 (11.3–11.4)	400,527	12.3 (12.3–12.4)	472,318	14.5 (14.5–14.6)	<0.0001
	60–69	321,544	17.0 (17.0–17.1)	341,361	18.1 (18.0–18.1)	363,451	19.2 (19.2–19.3)	429,228	22.7 (22.6–22.8)	<0.0001
	70–79	171,270	15.8 (15.7–15.9)	190,251	17.6 (17.5–17.6)	216,713	20.0 (19.9–20.1)	243,737	22.5 (22.4–22.6)	<0.0001
	80–89	30,830	11.9 (11.7–12.0)	35,106	13.5 (13.4–13.6)	41,127	15.8 (15.7–16.0)	48,910	18.8 (18.6–19.0)	<0.0001
Women	Total	1,072,510	5.7 (5.7–5.7)	1,135,578	6.1 (6.1–6.1)	1,201,116	6.4 (6.4–6.4)	1,473,739	7.9 (7.9–7.9)	<0.0001
	20–29	8,024	0.3 (0.2–0.3)	8,225	0.3 (0.3–0.3)	7,774	0.2 (0.2–0.3)	15,005	0.5 (0.5–0.5)	0.048
	30–39	30,799	0.8 (0.8–0.8)	30,816	0.8 (0.8–0.8)	30,974	0.8 (0.8–0.8)	50,097	1.3 (1.3–1.3)	0.481
	40–49	107,543	2.6 (2.6–2.6)	111,253	2.7 (2.7–2.7)	109,449	2.7 (2.7–2.7)	153,814	3.8 (3.7–3.8)	<0.0001
	50–59	230,093	6.9 (6.9–7.0)	243,840	7.4 (7.3–7.4)	256,632	7.7 (7.7–7.8)	318,061	9.6 (9.6–9.6)	<0.0001
	60–69	343,318	16.3 (16.3–16.4)	354,917	16.9 (16.8–16.9)	365,360	17.4 (17.3–17.4)	443,797	21.1 (21.0–21.2)	<0.0001
	70–79	281,703	18.0 (17.9–18.0)	304,826	19.5 (19.4–19.5)	334,111	21.3 (21.3–21.4)	382,547	24.4 (24.3–24.5)	<0.0001
	80–89	71,030	11.7 (11.6–11.8)	81,701	13.5 (13.4–13.6)	96,816	16.0 (15.9–16.1)	110,418	18.2 (18.1–18.3)	<0.0001

CI  =  confidence interval.

*P-values were obtained using Poisson regression.

The numbers of newly diagnosed T2DM patients in each year were 336,078, 331,387, and 321,966 in 2009, 2010, and 2011, respectively. The corresponding annual incidence rates of T2DM were 9.8, 9.7, and 9.5 per 1,000 person-years (PY), respectively (**Table 2**). The annual incidence of T2DM decreased after adjusting for age and gender (*P*<0.0001). We subsequently analyzed the annual incidence rate according to gender and age groups. The incidence rates per 1,000 PY were 10.7, 10.6, and 10.4 for men and 9.0, 8.9, and 8.5 for women (**Table 2**). Men and women aged 20–49 years showed a decreasing rate of incidence from 2009 to 2011; the incidence of diabetes in individuals aged 40–49 years decreased from 11.4 to 10.6 (per 1,000 PY) in men and from 6.0 to 5.2 (per 1,000 PY) in women (*P*<0.0001 in both).

**Table 2:** The incidence of type 2 diabetes mellitus in the Korean population aged 20–89 years.

		Year								
		2009		2010		2011		2009–2011		
		N	Incidence per 1,000 PY (95% CI)	N	Incidence per 1,000 PY (95% CI)	N	Incidence per 1,000 PY (95% CI)	N	Incidence per 1,000 PY (95% CI)	*P*-value*
Total		336,078	9.8 (9.8–9.8)	331,387	9.7 (9.7–9.7)	321,966	9.5 (9.4–9.5)	989,431	9.7 (9.6–9.7)	<.0001
Men	Total	177,999	10.7 (10.6–10.7)	175,808	10.6 (10.5–10.6)	172,967	10.4 (10.4–10.5)	526,774	10.6 (10.5–10.6)	<.0001
	20–29	2,931	0.9 (0.8–0.9)	2,748	0.8 (0.8–0.8)	2,622	0.8 (0.7–0.8)	8,301	0.8 (0.8–0.8)	<.0001
	30–39	16,609	4.3 (4.2–4.4)	15,445	4.0 (3.9–4.1)	15,216	3.9 (3.9–4.0)	47,270	4.1 (4.0–4.1)	<.0001
	40–49	44,215	11.4 (11.3–11.5)	41,628	10.8 (10.7–10.9)	40,838	10.6 (10.5–10.7)	126,681	10.9 (10.9–11.0)	<.0001
	50–59	52,070	18.1 (17.9–18.2)	52,952	18.6 (18.4–18.7)	53,702	19.0 (18.9–19.2)	158,724	18.6 (18.5–18.6)	<.0001
	60–69	38,778	25.0 (24.8–25.3)	38,627	25.3 (25.0–25.5)	36,400	24.1 (23.9–24.4)	113,805	24.8 (24.7–25.0)	<.0001
	70–79	19,482	21.6 (21.3–21.9)	20,331	23.0 (22.7–23.3)	20,104	23.5 (23.1–23.8)	59,917	22.7 (22.5–22.9)	0.002
	80–89	3,914	17.2 (16.7–17.8)	4,077	18.3 (17.7–18.8)	4,085	18.8 (18.3–19.4)	12,076	18.1 (17.8–18.4)	0.057
Women	Total	158,079	9.0 (9.0–9.0)	155,579	8.9 (8.8–8.9)	148,999	8.5 (8.5–8.6)	462,657	8.8 (8.8–8.8)	<.0001
	20–29	3,275	1.0 (1.0–1.1)	2,889	0.9 (0.9–0.9)	2,606	0.8 (0.8–0.9)	8,770	0.9 (0.9–0.9)	<.0001
	30–39	9,529	2.5 (2.4–2.5)	9,653	2.5 (2.5–2.6)	8,840	2.3 (2.3–2.4)	28,022	2.4 (2.4–2.5)	<.0001
	40–49	23,621	6.0 (5.9–6.0)	22,463	5.7 (5.6–5.7)	20,762	5.2 (5.2–5.3)	66,846	5.6 (5.6–5.7)	<.0001
	50–59	40,022	13.1 (12.9–13.2)	40,481	13.3 (13.1–13.4)	40,424	13.3 (13.2–13.4)	120,927	13.2 (13.1–13.3)	0.157
	60–69	42,119	24.2 (24.0–24.4)	40,756	23.6 (23.3–23.8)	37,756	22.0 (21.7–22.2)	120,631	23.2 (23.1–23.4)	<.0001
	70–79	30,783	24.2 (24.0–24.5)	30,587	24.5 (24.3–24.8)	29,938	24.6 (24.3–24.9)	91,308	24.5 (24.3–24.6)	0.001
	80–89	8,730	16.4 (16.1–16.8)	8,750	16.8 (16.5–17.2)	8,673	17.2 (16.8–17.5)	26,153	16.8 (16.6–17.0)	0.666

PY  =  person-years; CI  =  confidence interval.

* P-values were obtained using Poisson regression.

### Prevalence of macrovascular complication in the 2009 cohort

A total of 1,233,009 men and 1,135,578 women were defined as T2DM patients in 2009 (Cohort I). Among them, 177,999 men and 158,079 women were newly diagnosed as T2DM in that year (Cohort II). In Cohort I, the prevalence of CAD during the three-year period was 16.82%; among these individuals, 11.2% (N = 45,488, 1.92% of Cohort I) and 1.00% (N = 3,984, 0.17% of Cohort I) underwent percutaneous coronary intervention (PCI) and coronary artery bypass graft surgery (CABG), respectively (**Table 3**). As a marker of PAD incidence, 12,282 (0.52% of Cohort I) and 1,162 (0.05% of Cohort I) underwent percutaneous or open revascularization therapy, respectively, which corresponded to about 27% of the number of interventions for CAD during the same period. The amputation of lower extremities was detected in 6,891 (0.29% of Cohort I). CVD was detected in 11.38% of Cohort I from 2009–2011.

**Table 3:** The prevalence of diabetic macrovascular complications in patients with type 2 diabetes mellitus aged 20–89 years.

Year		N (%)							
		Pre-existing cases of T2DM in 2009 (N = 2,368,587, Cohort I)				New cases of T2DM in 2009 (N = 336,078, Cohort II)			
		2009	2010	2011	2009–2011	2009	2010	2011	2009–2011
CAD	Total*	241,924 (10.21)	244,348 (10.32)	244,988 (10.34)	398,389 (16.82)	29,365 (8.74)	26,444 (7.87)	26,067 (7.76)	47,211 (14.05)
	PCI	17,026 (0.72)	15,850 (0.67)	15,973 (0.67)	45,488 (1.92)	2,362 (0.70)	1,377 (0.41)	1,278 (0.38)	4,641 (1.38)
	Coronary bypass	1,583(0.07)	1,253 (0.05)	1,152 (0.05)	3,984 (0.17)	206 (0.06)	77 (0.02)	91 (0.03)	373 (0.11)
CVD	Total*	159,698 (6.74)	158,704 (6.70)	157,526 (6.65)	269,538 (11.38)	18,458 (5.49)	16,876 (5.02)	16,305 (4.85)	29,412 (8.75)
PAD	Percutaneous angioplasty	4,067 (0.17)	3,867 (0.16)	4,348 (0.18)	12,282 (0.52)	300 (0.09)	236 (0.07)	236 (0.07)	772 (0.23)
	Open revascularization	480 (0.02)	404 (0.02)	327 (0.01)	1,162 (0.05)	74 (0.02)	38 (0.01)	26 (0.01)	126 (0.04)
Amputation	Total	2,703 (0.11)	2,418 (0.10)	2,408 (0.10)	6,891 (0.29)	280 (0.08)	147 (0.04)	120 (0.04)	512 (0.15)
	Femur	147 (0.01)	131 (0.01)	118 (0.00)	386 (0.02)	26 (0.01)	15 (0.00)	12 (0.00)	53 (0.02)
	Below knee	729 (0.03)	645 (0.03)	603 (0.03)	1,914 (0.08)	71 (0.02)	39 (0.01)	22 (0.01)	131 (0.04)
	Foot	502 (0.02)	434 (0.02)	479 (0.02)	1,367 (0.06)	43 (0.01)	23 (0.01)	12 (0.00)	77 (0.02)
	Toe	1,722 (0.07)	1,550 (0.07)	1,579 (0.07)	4,529 (0.19)	175 (0.05)	90 (0.03)	84 (0.02)	329 (0.10)

T2DM  =  type 2 diabetes mellitus; CAD  =  coronary artery disease; CVD  =  cardiovascular disease; PAD  =  peripheral artery disease; PCI  =  percutaneous coronary intervention.

*Claim with an ICD-10 diagnosis code as the principal or additional diagnosis, or claims involving a procedure related to the corresponding disease.

In Cohort II, the prevalence of CAD and CVD from 2009–2011 was 14.05% and 8.75%, respectively, which were lower rates than those in Cohort I (**Table 3**). Percutaneous and open revascularization for PAD was performed in 772 (0.23% of Cohort II) and 126 (0.04% of Cohort II) subjects, respectively; and the amputation of lower extremities was detected in 512 (0.15%) subjects in Cohort II from 2009–2011.

Next, we analyzed the incidence of macrovascular complications in Cohort II. Among 336,078 subjects in Cohort II, 21,634 (mean days of follow-up ± standard deviation, 401±283 days) and 13,430 (408±280 days) subjects were newly diagnosed with CAD and CVD, respectively, from 2009–2011 (**Table 4**). Among these individuals, 6,107 and 4,510 were admitted for the management of CAD and CVD, respectively. The annual incidence rates of CAD per 1,000 PY in Cohort II were 18.84 in 2009, 25.71 in 2010, and 21.98 in 2011. About 30% of subjects diagnosed with CAD underwent admission for management of CAD (**Table 4**). The annual incidence rates of PCI for CAD per 1,000 PY in Cohort II were 1.58 in 2009, 2.43 in 2010, and 2.44 in 2011, while the annual incidence rates of percutaneous and open revascularization for PAD were 0.4, 0.6, and 0.7 during the same time period. From 2009–2011, 2,292 and 533 subjects in Cohort II underwent angioplasty for CAD and PAD, respectively. Among them, 78 subjects received angioplasty for both CAD and PAD. The annual incidence rates of admission for CVD per 1,000 PY were 3.83, 5.02, and 4.65 in 2009, 2010, and 2011, respectively.

**Table 4:** The incidence of diabetic macrovascular complications in Cohort II.

		Incidence, N (/1,000 person-year) (N = 336,078)								
Year		2009		2010		2011		2009–2011		Mean days of follow-up (SD)
CAD	Total*	6,274	(18.84)	8,373	(25.71)	6,987	(21.98)	21,634	(22.17)	400.8 (283.2)
	PCI	531	(1.58)	816	(2.43)	817	(2.44)	2,164	(2.15)	439.9 (287.6)
	Coronary bypass	37	(0.11)	45	(0.13)	63	(0.19)	145	(0.14)	463.5 (301.1)
	Admission†	1,725	(5.15)	2,293	(6.88)	2,089	(6.31)	6,107	(6.11)	409.4 (291.0)
CVD	Total*	3,782	(11.32)	5,189	(15.74)	4,459	(13.73)	13,430	(13.59)	408.3 (279.7)
	Admission†	1,286	(3.83)	1,677	(5.02)	1,547	(4.65)	4,510	(4.50)	414.9 (285.6)
PAD	Percutaneous angioplasty	108	(0.32)	183	(0.54)	205	(0.61)	496	(0.49)	453.0 (291.7)
	Open revascularization	24	(0.07)	19	(0.06)	14	(0.04)	57	(0.06)	320.1 (243.6)
Amputation	Total	160	(0.48)	126	(0.4)	102	(0.30)	388	(0.39)	340.3 (293.7)
	Femur	18	(0.05)	13	(0.04)	11	(0.03)	42	(0.04)	317.7 (270.1)
	Below knee	39	(0.12)	37	(0.11)	21	(0.06)	97	(0.10)	341.9 (286.2)
	Foot	26	(0.08)	23	(0.07)	11	(0.03)	60	(0.06)	303.8 (277.4)
	Toe	99	(0.29)	78	(0.23)	73	(0.22)	250	(0.25)	353.0 (304.1)

CAD  =  coronary artery disease; CVD  =  cardiovascular disease; PAD  =  peripheral artery disease; PCI  =  percutaneous coronary intervention; SD  =  standard deviation.

*Claim with an ICD-10 diagnosis code as the principal or additional diagnosis, or claims involving a procedure related to the corresponding disease.

† Hospital admissions were measured using inpatient claims with the principal diagnosis of the corresponding disease.

## Discussion

Our retrospective cohort study evaluated the incidence and prevalence of T2DM and associated macrovascular complications in Korea using the NHI HIRA database, which includes information for about 97% of Koreans. The prevalence of T2DM in Korean adults aged 20–89 years ranged from 6.1–6.9% from 2008–2010, and the annual incidence rate of T2DM ranged from 9.5–9.8/1,000 PY from 2009–2011. Women aged <60 years showed a lower prevalence of T2DM compared to men of the same age, and women between the ages of 30 and 49 years demonstrated about half the prevalence and incidence rate of men of the same age.

The incidence of T2DM in our study is similar to a previous report in Taiwan, that used diagnosis code-based claim data to show that the incidence was 7.8/1,000 PY in 2004. In Japan, a recent pooled analysis showed that the incidence rate of T2DM was 8.8/1,000 PY, which is also similar to our results [21]. Given that the populations of countries adjacent to Korea such as China and Japan have similar genetic backgrounds related to the risk of developing T2DM [22]–[24], and that such countries have similar T2DM prevalence [5], [6], [8], [25], our results for the Korean population are reasonable.

However, claim-based diagnoses may underestimate the real prevalence or incidence of T2DM. The mean annual incidence rate of T2DM in a Korean community-based cohort was 21.5/1,000 PY in adults aged 40–69 years [15], [26], which is higher than our results. The prevalence of T2DM in adults aged ≥30 years in 2010, according to the KNHANES 2010 was 10.1%, which is also higher than our result [13]. The consideration that only 73.0% of diabetic subjects were aware of their glucose disorder in 2008–2010 according to the KNHANES [13] may explain these differences explained. A previous national survey of diabetes in Korea based on 2003 HIRA data showed an incidence rate of diabetes of 5.7/1,000 PY, which is lower than our results [27]. As the proportion of diabetic subjects with a medical diagnosis of diabetes increased from 43% in 2001 and 66.5% in 2005 [28] to 73.0% in 2008–2010, according to the KNHANES [13], misclassification bias may account for a potential underestimation in the incidence of diabetes based on claims data in 2003 compared with data from 2009–2011.

Interestingly, the incidence of T2DM in men and women aged 20–49 years showed a decrease from 2009 to 2011, despite an increase in the prevalence of T2DM in the overall population. The incidence of diabetes in individuals aged 40–49 years decreased from 11.4 to 10.6/1,000 PY in men and from 6.0 to 5.2/1,000 PY in women, which corresponds to previous data from the KNHANES showing that the prevalence of diabetes among women aged 30–59 years decreased from 2001 to 2010 [14]. Lifestyle improvements such as decreasing total daily energy intake and performing regular exercise along with decreased rates of obesity in young adults in Korea, may be the causes of these trends [14]. In contrast, both the incidence and the prevalence of T2DM in individuals aged ≥70 years increased in our study. Korea is one of most rapidly aging countries, similar to Japan; the proportion of the elderly population aged ≥65 in Korea increased from 7.2% of the total population in 2000 to 11.0% in 2010 [29]. Our study showed that the incidence of T2DM in individuals aged 70–79 years was as high as that in individuals aged 60–69 years; furthermore, in women, individuals aged 70–79 years showed a higher incidence rate than those aged 60–69 years, which is worth noting. In general, the incidence of diabetes increases with age until about 65 years of age, after which both incidence and prevalence seem to decrease [30], [31]. We cannot explain the cause of the relatively high incidence of newly detected T2DM in individuals aged ≥70 years; further studies characterizing the elderly population with incident T2DM should be performed because increasing rates of T2DM among these individuals may increase the economic burden in Korea.

The prevalence of CAD and CVD in diabetic subjects in our study ranged from 10.2–10.3% and 6.7%, respectively, which was more than 2 times higher than the general population. In the 2010 KNHANES, the prevalence of CAD and CVD in adults aged ≥30 years was 2.4% and 1.4%, respectively [13]. Previous studies based on the NHI system in the general Korean population also showed a lower prevalence of both CAD and CVD (2.5% and 2.4%, respectively) [32], [33]. Comparing the newly diagnosed T2DM patients (Cohort II) with all T2DM patients (Cohort I), the prevalence of CAD and CVD was lower in Cohort II than in Cohort I. However, in Cohort II, up to 8.7% and 5.5% of Cohort II had CAD and CVD, respectively, in the year of T2DM diagnosis, which are still higher rates than those in the general Korean population.

The incidence of CAD and CVD in newly detected T2DM patients (Cohort II) was 18.84/1,000 PY and 11.32/1,000 PY in the year of T2DM diagnosis; these increased in subsequent years. The incidence of CAD was 25.71/1,000 PY in 2010 and 21.98/1,000 PY in 2011, and the incidence of CVD was 15.74/1,000 PY in 2010 and 13.73/1,000 PY in 2011. The incidence of ischemic stroke (resulting from CVD) in the general population of Korea was reported as 1.3/1,000 PY in 2004 [34].

The number of claims for PAD was relatively small compared to CAD and CVD. However, the finding that, among the 533 subjects in Cohort II who underwent angioplasty for PAD during study period, 78 (14.6%) of them experienced angioplasty for CAD during the same period emphasizes the importance of screening for CAD in T2DM patients with PAD considering the morbidity and mortality from CAD [35].

We confirmed the relatively higher risk of CAD and CVD in diabetic subjects compared to the general Korean population using nationwide health insurance claim data from HIRA. However, there are limitations regarding the accuracy of the diagnoses from claim data, since they were not based on clinical data. Furthermore, claims data provide limited information on disease severity, co-morbid conditions, past history, and specific treatment. As previously mentioned, the proportion of the population aware of their disease status may influence the estimation of prevalence or incidence of a disease in a claim study. In addition, three years of follow-up is a relatively short time period to evaluate trends in the incidence of T2DM. However, the usefulness of claim data in the nationwide survey for T2DM has been confirmed previously [36], [37]. The accuracy of diagnosis of T2DM from claim data reached a sensitivity of 68–71% and positive predictive value of 85–88% for clinically diagnosed T2DM from health examinations [37]. In addition, we extensively reviewed two years of prior claim data to calculate the incidence of T2DM, which may result in reasonable incidence rates compared to those from adjacent Asian populations and previous reports in Korea.

In summary, in recent years, the prevalence of T2DM in Korean adults aged 20–89 years was 6.1–6.9% and the annual incidence rate of T2DM was 8.8–9.2/1,000 PY. The fact that the incidence of T2DM in the elderly population increased significantly in the opposite direction to that in young men and women in the same period may result in an increasing economic burden in Korea, and necessitates a call for public health planning for the elderly population. Even in newly detected T2DM subjects, the prevalence of CAD and CVD was much higher than that in the general population. Our extensive investigation of epidemiologic data from nationwide claim data should be invaluable for planning national public health strategies.

## Supporting Information

File S1 **Supporting tables.**
(DOCX)
