# Supplementary material for: The Incidence and Prevalence of Diabetes Mellitus and Related Atherosclerotic Complications in Korea: A National Health Insurance Database Study
Source: PLoS One. 2014 Oct 16;9(10):e110650. doi: 10.1371/journal.pone.0110650 (PMC4199756; doi:10.1371/journal.pone.0110650)
Supplement: File S1 — Supporting tables. (DOCX) [file pone.0110650.s001.docx]

Table S1. Definition of diabetic macrovascular disease by ICD-10 codes and procedure/surgery in claim data

| **CAD** | **Diagnosis or procedure code** |
| --- | --- |
| Diagnosis | Ischemic heart disease (I20-5); cardiovascular disease (I98.8) |
| Percutaneous procedure* | Percutaneous transluminal coronary angioplasty (M6551, M6552)  Percutaneous transcatheter placement of intracoronary stent (M6561-4)  Percutaneous transluminal coronary atherectomy (M6571-2)  Percutaneous mechanical thrombolysis (M6633)  Percutaneous thrombolytic treatment, coronary artery (M6634)  Percutaneous intravascular atherectomy (M6620)^†^ |
| Coronary bypass surgery* | Vascular bypass operation, aorta-coronary (O1641, O1642, O1647 , OA641, OA642, OA647) |
| **CVD** | **Diagnosis or procedure code** |
| Diagnosis | Subarachnoid(I60), intracerebral (I61), and other nontraumatic intracranial (I62) hemorrhage |
|  | Cerebral infarction (I63); stroke, not specified as hemorrhage or infarction (I64) |
|  | Other cerebrovascular syndrome (G46.8) |
| Percutaneous procedure* | Percutaneous thrombus removal, cerebral (M6631)  Percutaneous transluminal angioplasty, cerebral and carotid (M6593-4)  Percutaneous cerebral angioplasty with drug (M6599)  Percutaneous intravascular installation of metallic stent cerebral and carotid (M6601-2) |
| **PAD** | **Diagnosis or procedure code** |
| Percutaneous Angioplasty* | Percutaneous transluminal angioplasty (M6597)  Percutaneous intravascular installation of metallic stent (M6605)  Percutaneous intravascular installation of stent-graft (M6613)  Percutaneous thrombus removal (M6632)  Percutaneous intravascular atherectomy (M6620)^‡^ |
| Open revascularization* | Vascular bypass operation, artery, others (O1643-4, O0161~O0171, O1645~6)^‡^ |
| (Diagnosis) | Atherosclerosis of upper/lower limb (I70.2-3); atherosclerosis, unspecified (I70.9); thromboangiitis obliterans (I73.1); peripheral vascular disease (I73.8-9) |
| **Amputation** | **Procedure code** |
| Amputation^*^ | Amputation of femur (N0572) |
|  | Amputation below knee (N0573) |
|  | Amputation of foot (N0574) |
|  | Amputation of toe (N0575) |

ICD-10 = International Classification of Diseases (10th revision); CAD = coronary artery disease, CVD = cardiovascular disease; PAD = peripheral artery disease.

^*^Healthcare Common Procedure Coding System codes provided by HIRA; ^†^Procedure under diagnosis code of [I20 - I25]; ^‡^ Procedure under diagnosis code of [I70.2 - 3, I70.9, I73.1, I73.8-9]

Table S2. Korean population data from the 2010 Population and Housing Census

|  | **Total** | **Men** | **Women** |
| --- | --- | --- | --- |
| Total | 47,990,761 | 23,840,896 | 24,149,865 |
| 0 – 4 years | 2,219,084 | 1,142,220 | 1,076,864 |
| 5 – 9 years | 2,394,663 | 1,243,294 | 1,151,369 |
| 10 – 14 years | 3,173,226 | 1,654,964 | 1,518,262 |
| 15 – 19 years | 3,438,414 | 1,826,179 | 1,612,235 |
| 20 – 24 years | 3,055,420 | 1,625,371 | 1,430,049 |
| 25 – 29 years | 3,538,949 | 1,802,805 | 1,736,144 |
| 30 – 34 years | 3,695,348 | 1,866,397 | 1,828,951 |
| 35 – 39 years | 4,099,147 | 2,060,233 | 2,038,914 |
| 40 – 44 years | 4,131,423 | 2,071,431 | 2,059,992 |
| 45 – 49 years | 4,073,358 | 2,044,641 | 2,028,717 |
| 50 – 54 years | 3,798,131 | 1,887,973 | 1,910,158 |
| 55 – 59 years | 2,766,695 | 1,360,747 | 1,405,948 |
| 60 – 64 years | 2,182,236 | 1,057,035 | 1,125,201 |
| 65 – 69 years | 1,812,168 | 833,242 | 978,926 |
| 70 – 74 years | 1,566,014 | 672,894 | 893,120 |
| 75 – 79 years | 1,084,367 | 410,726 | 673,641 |
| 80 – 84 years | 595,509 | 186,008 | 409,501 |
| 85 – 89 years | 271,166 | 74,118 | 197,048 |
| 90 – 94 years | 78,329 | 17,770 | 60,559 |
| 95 – 99 years | 15,279 | 2,593 | 12,686 |
| ≥100 years | 1,835 | 255 | 1,580 |
